# Management of the positive sentinel lymph node following neoadjuvant chemotherapy: results of a survey conducted with breast surgeons

**DOI:** 10.3332/ecancer.2022.1357

**Published:** 2022-02-18

**Authors:** Francisco Pimentel Cavalcante, Felipe Zerwes, Eduardo Camargo Millen, Guilherme Novita, Alessandra Borba Anton de Souza, João Henrique Penna Reis, Helio Rubens de Oliveira Filho, Luciana Naíra de B L Limongi, Barbara Pace Silva de Assis Carvalho, Adriana Magalhães de Oliveira Freitas, Monica Travassos Jourdan1, Vilmar Marques de Oliveira, Ruffo Freitas-Junior

**Affiliations:** 1 Breast Unit, Fortaleza General Hospital (HGF), Fortaleza, CE 60150160, Brazil; 2 School of Medicine, Pontificia Universidade Católica do Rio Grande do Sul (PUCRS), Porto Alegre, RS 90450130, Brazil; 3 Instituto Oncoclinicas, Rio de Janeiro, RJ 22440040, Brazil; 4 Breast Unit, Hospital Israelita Albert Einstein, São Paulo, SP 01321001, Brazil; 5 Oncology Research Group-CNPq, PUCRS, Porto Alegre, RS 90610000, Brazil; 6 Breast Center, Instituto Orizonti and Hospital Mater Dei, Belo Horizonte, Minas Gerais, MG 30210080, Brazil; 7 School of Medicine, Federal University of Paraná, Curitiba, PR 80060900, Brazil; 8 Breast Unit, Real Hospital Português, Recife, PE 52010075, Brazil; 9 Breast Unit, Pace Hospital, Belo Horizonte, MG 30110062, Brazil; 10 Breast Unit, Larmony Mastologia, Florianópolis, SC 88015300, Brazil; 11 Breast Unit, Samaritano Botafogo (Américas Serviços Médicos), Rio de Janeiro, RJ 22270010, Brazil; 12 School of Medical Sciences, Santa Casa de São Paulo, São Paulo, SP 01224001, Brazil; 13 Breast Unit, Santa Casa de São Paulo, São Paulo, SP 01221010, Brazil; 14 CORA Advanced Centre for Diagnosis of Breast Cancer, Department of Obstetrics and Gynaecology, Federal University of Goiás, Goiânia, GO 74605050, Brazil; a https://orcid.org/0000-0002-7156-2890; b https://orcid.org/0000-0002-1643-727X; c https://orcid.org/0000-0002-2113-6324; d https://orcid.org/0000-0003-2983-3199; e https://orcid.org/0000-0002-6215-7076; f https://orcid.org/0000-0002-7754-9793; g https://orcid.org/0000-0003-4136-7047; h https://orcid.org/0000-0001-9014-4793; i https://orcid.org/0000-0003-1888-4433; j https://orcid.org/0000-0003-1068-4154; k https://orcid.org/0000-0003-0910-0270; l https://orcid.org/0000-0002-9478-5616; m https://orcid.org/0000-0003-4145-8598

**Keywords:** breast cancer, neoadjuvant chemotherapy, residual nodal disease, sentinel lymph node biopsy, axillary dissection, regional nodal irradiation

## Abstract

**Introduction:**

Despite the lack of randomised evidence, there is a current trend towards omitting axillary surgery in cases of positive sentinel lymph node (SLN) following neoadjuvant chemotherapy (NACT). This study evaluated practice patterns of Brazilian breast surgeons when managing positive SLN following NACT.

**Methods:**

This was a nationwide electronic survey of breast surgeons affiliated with the Brazilian Society of Mastology. Management approaches for positive SLN after NACT (axillary dissection (AD), regional nodal irradiation (RNI) or no additional treatment) were evaluated as a function of residual disease volume in the SLN (macro-metastasis, micro-metastasis or isolated tumour cells (ITC)).

**Results:**

Survey response rate was 49%, with 799/1,627 questionnaires returned. Most respondents were <50 years old (61%), lived in south-eastern Brazil (50%), in a major city (67%), worked in an academic institute (80%) and were board-certified (80%). AD recommendation rate decreased according to residual nodal disease volume: 91% of respondents recommended AD for cases of macro-metastasis, 64% for micro-metastasis and 38% for ITC (*p* < 0.00001). Furthermore, 35% would recommend no additional surgery for micro-metastasis, while 27% would recommend no treatment at all for ITC (*p* < 0.00001). Not working in an academic institute was associated with RNI for micro-metastasis (*p* = 0.02), but not for macro-metastasis or ITC. Being board-certified did not affect axillary management.

**Conclusion:**

Most respondents would recommend AD and/or RNI in residual nodal disease following NACT irrespective of disease volume. Nevertheless, a trend towards surgical de-escalation was found with low-volume disease (micro-metastasis and ITC). Ongoing randomised trials will clarify the impact of this trend.

## Introduction

Sentinel lymph node (SLN) biopsy is the approach of choice in axillary surgery for patients with early breast cancer and clinically negative axilla [1, 2]. The technique provides excellent regional control compared to axillary dissection (AD) and a lower rate of lymphedema. Currently, AD has also been safely omitted during upfront surgery in cases in which there is only limited disease in the SLN [3–6]. The possibility of reducing the extent of axillary surgery has been evaluated in different situations in order to decrease rates of surgical morbidity while still guaranteeing accurate staging information, which is crucial for the planning of adjuvant treatment [7–9].

Systemic neoadjuvant chemotherapy (NACT), traditionally used in locally advanced breast cancer to facilitate breast-conserving surgery, has now been recommended to enable downstaging in clinically positive axillae. This strategy has shown acceptable false-negative rates comparable to upfront surgery with the resection of three or more negative SLN or the removal of the previously clipped node [7–11]. Nevertheless, omitting AD of positive SLN following NACT remains a much-debated issue.

An ongoing randomised clinical trial sponsored by the Alliance for Clinical Trials in Oncology [12] is evaluating the possibility of substituting AD for axillary radiation in patients with positive SLN following NACT. The concept that low-volume disease in the SLN can respond well to the local treatment either with radiotherapy or AD without involving any significant difference in overall survival (OS) was well documented in the AMAROS (After Mapping of the Axila: Radiotherapy or Surgery) trial [5]. However, randomised clinical trial data are still expected to clarify whether this concept is also valid in cases of residual low-volume disease in the SLN after NACT. The debate remains open on whether this state of disease could lead to a poorer prognosis and an increased likelihood of local recurrence, ultimately requiring AD. Until the results of the currently ongoing randomised clinical trial on this subject are available, the relevant breast societies still recommend AD if residual axillary disease after NACT is detected [12–14].

Regardless of these recommendations following NACT, in recent years, there has been a growing trend towards omitting additional axillary surgery despite the lack of evidence from randomised clinical trials focusing on this particular clinical endpoint. In routine clinical practice, breast surgeons are sometimes confronted with different situations such as when SLN biopsy is negative on frozen section but found to be positive/metastatic on paraffin section, with the surgeon then making the decision not to return the patient to the operating room to re-operate the axilla [15–18]. Indeed, although guidelines recommended AD if there is any residual disease after NACT, in practice, if residual disease is not detected at the time of frozen section biopsy but only in the definitive result, regional nodal irradiation (RNI) without AD can be considered by the multidisciplinary team [14]. Therefore, specific surveys on this subject are of the utmost importance in understanding what occurs in real world practice.

The principal objective of this study was to evaluate current trends among breast surgeons affiliated with the Brazilian Society of Mastology (SBM) with respect to their approach to axillary surgery following NACT with a positive SLN, particularly based on nodal disease volume.

## Materials and methods

This is the second part of a nationwide electronic survey conducted between 25 June and 24 August 2020 with 1,627 breast surgeons affiliated with the SBM. While the first part of this survey referred to the management of positive SLN in upfront surgery (results in press), the current analysis refers to the management of the axilla in cases of positive SLN after NACT, in the presence of macro-metastasis, micro-metastasis or isolated tumour cells (ITC) in the SLN.

The SBM membership criteria require medical residency and/or board-certification in breast diseases. In Brazil, there is a specific medical residency program aimed at training specialists in breast surgery. Most of the SBM members work primarily on the treatment of breast cancer and most are board-certified by the Brazilian Medical Association. In this study, all the participants were specialists in breast cancer surgery and members of the SBM, although in some cases their specialist diploma had not been board-certified by the SBM itself.

The SBM provided data on the age and sex of its members, the region in which they worked, and whether or not they were board-certified. This allowed those who completed and returned the questionnaire to be compared with the group of non-respondents. The SBM adopts the international guideline recommendations for the technique of SLN biopsy. However, since most of the oncology institutes in the country do not have access to nuclear medicine services, blue dye alone tends to be used as a lymph node marker for the majority of patients [16, 17].

The survey contained questions aimed at obtaining participants’ demographic data: age, sex, the region in which they worked (southeast, northeast, south, north or mid-west of the country), whether they were board-certified in breast surgery and whether or not they worked in an academic institute. For each category of disease volume in the SLN (macro-metastasis, micro-metastasis or ITC), there were three possible answers: AD, RNI or no additional treatment. According to the Tumor size, Lymph Nodes, Metastasis (TNM) classification of malignant tumours, macro-metastasis was defined as >2 mm in the SLN, micro-metastasis as ≤2 and >0.2 mm, and ITC as ≤0.2 mm. The survey questionnaire is shown in Figure 1.

The internal review board of the SBM approved the study protocol and waived the requirement for informed consent since the questionnaires were to be answered anonymously. The data were analysed using Statistical Package for the Social Sciences, version 26.0. The demographic profile of the study sample and the management approach used by the respondents were evaluated using measures of absolute (*n*) and relative frequency (%). Relative frequencies were analysed according to the number of participants answering each individual question. The association between demographic characteristics and the management approach adopted was analysed using contingency tables and Pearson’s post-hoc chi-square test. Significance was set at 5% (*p* < 0.05).

## Results

Of the 1,627 questionnaires sent out to SBM members, 799 were completed and returned, resulting in a response rate of 49%. Of the respondents, 61% were under 50 years of age, 49% were female, and 80% were board-certified breast surgeons. In relation to their place of work, 80% worked in an academic institute or in an institute working exclusively with breast cancer. Most (67%) lived in a state capital city and 50% lived in the southeast of the country compared to 21% in the northeast, 14% in the south, 9% in the mid-west and 3% in the north of Brazil (Table 1). The group of surgeons who answered the questionnaire was similar to the group of non-respondents in terms of sex (*p* = 0.13), the region of the country in which they lived/worked (*p* = 0.99) and board-certification (*p* = 13). The only significant difference between the two groups refers to a predominance of non-respondents in the subgroup of individuals aged ≥70 years (*p* < 0.01) (Table 2).

For the purposes of describing axillary management when there is residual disease following NACT, three different possibilities of residual disease are considered here: macro-metastasis, micro-metastasis and isolated tumor cells, as already mentioned in the methods section. There are also three different options of possible axillary management: AD, RNI and no treatment.

If macro-metastasis were present in the SLN following NACT, 91% (*n* = 732) of respondents would recommend AD compared to 6% who would recommend RNI alone and 2% who would recommend no additional axillary treatment. In the case of low-volume disease in the SLN, however, a significant change was seen: in cases of micro-metastasis, 35% of surgeons would not recommend any additional surgery (*p* < 0.00001), with 26.5% recommending RNI and 8.6% no further additional local treatment in the axilla. The same was also found with ITC, with 61% of respondents not recommending AD (only 38% would recommend AD, while 34% would recommend RNI and 27% would recommend no further treatment) (Tables 3 and 4).

The extent of the treatment recommended decreased as a function of the volume of the disease. In cases in which ITC are present, 61% of respondents would recommend no surgical treatment compared to 35% if micro-metastasis is present and only 8% if there is macro-metastasis. For the purpose of analysis, RNI and no treatment have been grouped together as ‘no further surgical treatment’ (Table 4).

In the present study, not working in an academic institute was associated with recommending RNI in cases of micro-metastasis (*p* = 0.02); however, there was no such effect in cases of macro-metastasis (*p* = 0.14) or ITC (*p* = 0.05). On the other hand, whether the surgeon was board-certified had no effect on answers, irrespective of the volume of disease in the SLN (Table 5).

## Discussion

Most Brazilian breast surgeons participating in this survey would recommend AD in cases of residual disease in the SLN following NACT, particularly when involving macro-metastasis. A recent survey conducted with North American breast cancer providers on the extrapolation of axillary management to situations not covered by the Z0011 criteria, including patients treated with systemic NACT, found that 85% of respondents believed that more data would have to be available for clinical practice to change [18]. On the other hand, increased interest in omitting AD in such cases has been noted. One study, conducted using data from the National Cancer Database (NCDB), evaluated the trend for axillary surgery following NACT in patients with clinically node positive disease prior to and following publication of the Z0011 study. The rate of SLN biopsy alone increased from 25.6% in 2006 to 33.2% in 2012 in patients submitted to breast-conserving surgery (*p* < 0.01). That finding indicates that the results of the Z0011 in upfront surgery are being extrapolated to the situation of neoadjuvant treatment [15].

The Z0011 study confirmed the safety of omitting AD in patients with one or two positive SLNs in upfront surgery; however, that study excluded patients submitted to NACT [3]. There are no prospective, randomised studies on the oncologic safety of omitting AD in cases of residual disease in the SLN following NACT. A small retrospective study involving a short follow-up time in 161 patients with positive SLN after NACT detected no difference in regional control at 3 years, irrespective of the extent of axillary surgery; however, neither control of the disease nor survival was predicted [19]. The question remains whether AD would have an impact on the control of the disease or whether RNI could substitute it. A study conducted using data from the NCDB compared patients with positive SLN following NACT submitted to SLN dissection (removal of ≤4 lymph nodes) and RNI (*n* = 304) to a group submitted to AD and RNI (*n* = 1,313), following a design that was similar to that used in the ongoing ALLIANCE A011202 randomised, phase III, clinical trial [20]. Survival was poorer in the cases in which AD was omitted (HR = 1.7; 95% CI: 1.3–2.2; *p* < 0.001), with a 5-year survival rate of 71% compared to 77% in the AD group (*p* = 0.01). In that same study, an exploratory analysis showed SLN dissection to be comparable to AD in patients with hormone-positive tumours and with metastasis in a single lymph node, a finding more compatible with the results of the Z11 in upfront surgery. A retrospective study on patients receiving NACT between 2008 and 2013 in a single Korean institute involved only patients with one or two positive SLN following NACT who were followed up for a mean of 71 months. SLN biopsy alone (*n* = 98) was compared to AD (*n* = 98), with results showing no difference in OS (92.1% versus 91.1%; *p* = 0.809) [21]. Nevertheless, those studies could have involved biases with respect to lower-risk patients being selected for SLN biopsy alone, while recommending AD for those with greater residual nodal burden, thus affecting the results.

The presence of low-volume disease in the lymph node following NACT has also been a topic of debate. In the present analysis, 91% of the surgeons would recommend AD in cases of macro-metastasis; however, a trend was seen towards de-escalation with respect to axillary surgery in cases of micro-metastasis and ITC, with 35% and 61% of surgeons, respectively, not recommending any additional surgery. These findings could possibly reflect a belief that in cases of micro-metastasis and ITC following NACT the oncological outcome would be similar to that found in pathologically negative cases, as occurs in upfront surgery [19]. Historically, the presence of low-volume residual disease in the lymph node following NACT has generated controversy with respect to disease-free survival and OS. A retrospective review evaluated the burden of residual disease in positive SLN following NACT and showed a high additional tumour burden, irrespective of whether it consisted of micro-metastasis (59%) or macro-metastasis (63%), as a possible indicator for AD [22]. In another study conducted using the databases from the Dana-Farber/Brigham and Women’s Cancer Center and the NCDB, the presence of residual ITC and micro-metastasis was associated with poorer disease-free survival when compared to the absence of disease in the lymph node. That study concluded that following NACT, a low volume of residual disease implied a poorer prognosis compared to negative nodes, particularly in patients with triple-negative and HER2 tumours [23]. In fact, the burden of residual disease in the axilla following NACT and a positive SLN appears to be high, irrespective of tumour subtype [24]. On the other hand, a study of cancer records in the Netherlands involving 1,347 patients with an initially positive lymph node treated with NACT and submitted to AD showed comparable disease-free and OS rates in ypN0 and ypNitc/mic patients, but significantly different rates in ypN0 and ypN1-3 patients, with this latter group being associated with a less favourable prognosis [25]. Another study conducted with 98 patients submitted to AD following NACT and with residual disease in the SLN reported similar results in the group with micro-metastasis and in the group with negative SLN, with these groups having a more favourable outcome when compared to the cases involving macro-metastasis [26]. Again, the question is whether AD can be omitted in such cases. During the 2021 St. Gallen International Breast Cancer Consensus Conference, the topic of axillary surgery following NACT and residual disease in the SLN generated much debate. According to 73% of the panellists, AD should be recommended whenever macro-metastasis is identified; however, 60% opposed AD when micro-metastasis was present and 89% opposed any additional axillary surgery when the only finding in the SLN was ITC [27]. These results show a tendency towards de-escalation in axillary surgery, particularly in cases of low-volume disease in the SLN following NACT; however, the question of micro-metastasis was fiercely debated, showing that, indeed, the lack of randomised clinical trials on this issue still leaves many surgeons uncomfortable at omitting AD.

There are some limitations associated with the present study. Since it consists of a survey, it is impossible to guarantee that these results would be applicable in real life. The response rate of 49% is another limitation; however, there was no statistically significant difference between the group of surgeons who completed and returned the questionnaire and those who did not. The fact that no question was included in the survey on how to manage a positive SLN after neoadjuvant endocrine therapy (NET) may represent a further limitation. In our opinion, however, these are different circumstances compared to NACT, particularly in initially clinically node-negative (cN0) cases. In general, pathologic complete response following NET is not expected and the prognosis is therefore different [28]. A study conducted with data from the NCDB and from a single institute evaluated the burden of residual disease following NET and the type of axillary surgery performed: SLN biopsy or AD. More than 90% of the patients in both cohorts had cN0 axilla at the initial presentation and had fewer than three positive lymph nodes at final pathology, with no difference in OS irrespective of the type of axillary surgery [29]. This finding suggests that patients who are initially cN0 and SLN-positive following NET could be similar in profile to those of the Z0011 study; hence, AD could be omitted.

## Conclusions

The present results suggest that the majority of respondents would recommend AD or RNI when residual disease is identified in the SLN following NACT irrespective of the volume of residual disease in the lymph node. There was, however, a trend towards de-escalation of surgery in cases of low-volume disease in the SLN. Further studies are required to increase understanding of this type of case, and the results of randomised clinical trials on clinical outcomes will be crucial in optimising axillary surgery. While randomised evidence is awaited, surveys, real world data and survival analyses in cohort studies may contribute to improving understanding of this trend towards extrapolating conservative management in axillary surgery from upfront surgery studies to this scenario after NACT.

## Abbreviations

AD: Axillary dissection
ITC: Isolated tumour cells
NACT: Neoadjuvant chemotherapy
NET: Neoadjuvant endocrine therapy
RNI: Regional nodal irradiation
SBM: Brazilian Society of Mastology
SLN: Sentinel lymph node

## Conflicts of interest

The authors declare that they have no conflicts of interest.

## Funding

None.

## Authors’ contributions

**Francisco Pimentel Cavalcante, Felipe Zerwes, Eduardo Camargo Millen and Ruffo Freitas-Junior:** conceptualisation, methodology. **Francisco Pimentel Cavalcante, Felipe Zerwes, Eduardo Camargo Millen, Guilherme Novita, João Henrique Penna Reis, Alessandra Borba Anton de Souza and Ruffo Freitas-Junior:** formal analysis, investigation. **All authors:** data curation, writing - Original draft preparation, writing - reviewing and editing.

## Figures and Tables

**Figure 1.** Box showing the survey questionnaire and the options given as possible answers.

Questions		Possible answers				
Demographic characteristics						
1	How old are you?	(Open question)				
2	Sex	Female	Male			
3	Are you a board-certified breast specialist?	Yes	No			
4	Do you work in an institute that deals exclusively with cancer or in a treatment referral centre?	Yes	No			
5	In which region of Brazil do you work?	North	Northeast	Midwest	Southeast	South
6	Do you live in a state capital city?	Yes	No			
Management						
**7**	What is your approach in cases of a SLN with macro-metastasis (>2 mm) following NACT?	AD	RNI	No additional treatment		
8	What is your approach in cases of a SLN with micro-metastasis (>0.2 and ≤2 mm) following NACT?	AD	RNI	No additional treatment		
9	What is your approach in cases of a SLN with ITCs (≤0.2 mm) following NACT?	AD	RNI	No additional treatment		

**Table 1.** Demographic characteristics of the respondents.

Characteristics	*n*	%
Age group		
<50 years	493	61.7
≥50 years	265	33.2
Sex		
Female	394	49.3
Male	405	50.7
Board certification		
No	155	19.4
Yes	644	80.6
Works in an academic institute		
No	157	19.6
Yes	642	80.4
Region of residence		
Midwest	74	9.3
Northeast	172	21.5
North	27	3.4
Southeast	407	50.9
South	119	14.9
Lives in a state capital city		
No	257	32.2
Yes	542	67.8

**Table 2.** Demographic characteristics of respondents versus non-respondents.

Characteristics	Non-respondents (*n* = 828; 50.9%)		Respondents (*n* = 799; 49.1%)		Total (*n* = 1,627)		*p*-value^a^
	*n*	%	*n*	%	*n*	%	
Sex							0.13
Male	389	46.9	405	50.6	793	48.7	
Female	439	53.1	394	49.3	834	51.3	
Age group (years)^c^							<0.01
<30	16	2.0	14	1.8	30	1.9	
31–40	262	32.8	284	37.5	546	35.3	
41–50	208	26.0	217	28.6	425	29.2	
51–60	156	19.5	154	20.3	300	19.4	
61–70	87	10.9	67	8.8	154	9.9	
>70	68^b^	8.5	22	2.9	90	5.8	
Region of residence							0.99
Southeast	419	50.6	407	50.9	826	50.7	
Northeast	177	21.4	172	21.5	348	21.3	
South	123	14.8	119	14.9	243	14.9	
Midwest	80	9.7	74	9.3	154	9.4	
North	29	3.5	27	3.4	56	3.4	
Board certification							0.13
Yes	693	83.7	644	80.6	1341	82.4	
No	135	16.3	155	19.4	286	17.6	

a Pearson’s chi-square test

b Post hoc

c Data analysed as a function of the number of individuals who answered the question

**Table 3.** Management of the positive SLN following NACT according to nodal disease volume.

Management approach	Macro-metastasis	Micro-metastasis	ITCs
AD	732 (91.6%)	518 (64.8%)	311 (38.9%)
RNI	50 (6.3%)	212 (26.5%)	272 (34%)
No treatment	17 (2.1%)	69 (8.6%)	216 (27%)

**Table 4.** Surgical versus non-surgical management of positive SLN following NACT according to nodal disease volume.

Management approach^b^	Macro-metastasis	Micro-metastasis	ITCs
AD^b^	732 (91.6%)	518 (64.8%)	311 (38.9%)
Non-surgical approach^ab^	67 (8.3%)	281 (35.1%)	488 (61%)

a RNI or no further treatment at all

b Person’s chi-square test: *p*-value < 0.00001

SLN: Sentinel lymph node; NACT: Neoadjuvant chemotherapy

**Table 5.** Management of the positive SLN after NACT according to place of work and board-certification.

Management approach per nodal disease volume	No *n* (%)	Yes *n* (%)	*p*-value
Surgeon works in an academic institute			
Macro-metastasis			
RNI	**15 (9.6%)**	**35 (5.5%)**	*p* ** = 0.14**
AD	**138 (87.9%)**	**594 (92.5%)**	
No treatment	**4 (2.5%)**	**12 (2.0%)**	
Micro-metastasis			
RNI	**55 (35.0%)**	**157 (24.5%)**	***p* = 0.02**
AD	**88 (56.1%)**	**430 (67.0%)**	
No treatment	**14 (8.9%)**	**55 (8.6%)**	
ITCs			
RNI	**58 (36.9%)**	**214 (33.3%)**	*p* ** = 0.05**
AD	**48 (30.6%)**	**263 (41.0%)**	
No treatment	**51 (32.5%)**	**165 (25.7%)**	
Surgeon is board-certified			
Macro-metastasis			
RNI	**12 (7.7%)**	**38 (5.9%)**	*p* ** = 0.62**
AD	**139 (89.7%)**	**593 (92.1%)**	
No treatment	**4 (2.6%)**	**13 (2.0%)**	
Micro-metastasis			
RNI	**40 (25.8%)**	**172 (26.7%)**	*p* **= 0.88**
AD	**100 (64.5%)**	**418 (64.9%)**	
No treatment	**15 (9.7%)**	**54 (8.4%)**	
ITCs			
RNI	**55 (35.5%)**	**217 (33.7%)**	*p* ** = 0.37**
AD	**65 (41.9%)**	**246 (38.2%)**	
No treatment	**35 (22.6%)**	**181 (28.1%)**	

SLN: Sentinel lymph node; NACT: Neoadjuvant chemotherapy
